# Supplementary material for: A multilevel analysis of the predictors of health facility delivery in Ghana: Evidence from the 2014 Demographic and Health Survey
Source: PLOS Glob Public Health. 2024 Mar 13;4(3):e0001254. doi: 10.1371/journal.pgph.0001254 (PMC10936823; doi:10.1371/journal.pgph.0001254)
Supplement: Attachment — Submitted filename: Rebuttal letter R1.pdf [file pgph.0001254.s002.pdf]

A multilevel analysis of the predictors of health facility delivery in Ghana: evidence from the 2014 Demographic and Health Survey

POINT-BY-POINT RESPONSES TO EDITORS AND REVIEWERS

We wish to express our heartfelt thanks and appreciation to the Editors and the Reviewers for taking time of their tight schedule to review our manuscript and we found their comments very useful.

We have taken all the necessary steps to respond to your comments and thus, resubmit the revised manuscript for consideration and subsequent publication in your cherished journal.

The responses to your comments are presented below and the changes effected in the main manuscript for your consideration. Furthermore, minor changes have been made to the manuscript purposely to enhance the quality of the manuscript.

Journal Requirements:

1. Please provide additional details regarding participant consent. In the ethics statement, please ensure that you have specified what type you obtained (for instance, written or verbal, and if verbal, how it was documented and witnessed). If your study included minors, state whether you obtained consent from parents or guardians.
   Response: Thank you. We revised the manuscript to reflect this. See lines 359-361 at page 14.

Additional Editor Comments (if provided) (1):

Thank you for your submission, and we acknowledge the delays in review due to our challenges in finding suitable reviewers who agreed to this assignment. We have now met our target.
You will see detailed feedback from the reviewers. Of particular note are the concerns about the datedness of the data and the overlap with other work. The authors address some of these latter points in their paper, but this needs more discussion on the overlap and more emphasis on the unique contributions of this paper.
Response: Thank you. We revised the manuscript to focus on the need for multilevel rather than the individual predictors and addressed the overlap and uniqueness of this work. See lines 49-58, and lines 79-81 at page 3, 507-511 at page 27, lines 530-554, lines 571-581 at pages 28-29, lines 571-581 at page 30. Regarding the datedness of the data, this is the most recently available nationwide survey data in Ghana on this critical public health issue of global interests and our focus is on the Ghana DHS data because this is the most recent full DHS data available for Ghana presently. We revise the manuscript to indicate that the 2014 GDHS is the most recent full DHS data available for Ghana. See lines 227-228 at page 8. We have also included a statement in the limitation. See lines 681-682 at page 34. The data from Ghana DHS round conducted in late 2022 and early 2023 is yet to be released by the implementing agencies and therefore not available for use. We will update our models in our future research on this topic when the new DHS data is made available to the public.

Additional Editor Comments (2)

Thank you for your resubmission; I have read your responses closely and reread the manuscript. Given the delays in reviews from the first round, I want to make sure this is as tight as possible before sending to reviewers again. As such, please see my comments below. I kindly ask that: 1) you reply to these comments on their own; 2) that you resend your response to reviewers but make any updates to your responses to reflect changes based on these comments as well. (The reviewers will review your responses in the next phase.)

1) I noted your replies about the datedness of the data and the reasoning for this. Please update in

the manuscript that a) this is the most recent full DHS (presuming the 2017 DHS does not have sufficient sample size?); b) in your limitations, discuss why you think the results will or will not hold (or what results will and will not hold) the test of time? Will there be changes in the distributions in Table 1? Will the effect sizes of the independent variables be maintained? Is the influence of household or community variables still the same?
Response: Thank you. The 2014 DHS data is the most recent full DHS data in Ghana. The special survey conducted in 2017 is not a full DHS survey but Ghana Maternal Health Survey. However, in this study, our focus is on the Ghana DHS data because this is the most recent full DHS data available for Ghana presently. We revise the manuscript to indicate that the 2014 GDHS is the most recent full DHS data available for Ghana. See lines 227-228 at page 8. We have also included a statement in the limitation. See lines 681-682 at page 34. The data from Ghana DHS round conducted in late 2022 and early 2023 is yet to be released by the implementing agencies and therefore not available for use. We will update our models in our future research on this topic when the new DHS data is made available to the public.

2) I am not entirely convinced by your response about the use of the terms "geospatial" (in the paper or in your response). Specifically, what you are doing is using the coordinates of the respondents to identify the region to then calculate proportions with facility delivery. I would say this is GIS to calculate regional proportions and not "geospatial mapping" - from the reviewer, and I agree, you are making this seem more detailed than it really is. It also isn't clear that this adds anything to the paper. You don't put these proportions into the model, right? Could you have calculated this on a more granular level and included? Personally, if you aren't doing more with this data, then I suggest you drop this section. If you do keep, you need to make it more clear what this adds to the paper, be more specific on the techniques, and talk about how obfuscated coordinates where considered in these estimates.
Response: Thank you. We revised the manuscript by deleting this section and proving a summary in lines 349-351 at pages 13-14 and lines 238-244 at page 9.

3) Your discussion is very long, and in its length hides the key points. You are arguing the novelty of the paper, as compared to the many other papers looking at predictors of facility delivery in Ghana, is the multilevel modeling. This is where your discussion emphasis should be, rather than over discussion/interpretation of the specific individual parameters. Can you give a larger percent representation (discuss more, and discuss the other sections less) on: a) What can we say about HH and community level factors given the results of the 4 models? (and for this point, in the conclusion "intervening on HH and community factors" needs more specifics that are introduced earlier - what interventions? can we say that now or is more work needed?) and b) How does what we say about individual factors change when we account for these nested structure of the data? (I would argue from your results, not much. But this is totally lost in the hyper-interpretation of these effect sizes)
Response: Thank you for the useful comments. All the model metrics (AIC, Log(PL), AUROC value, and SE) strongly supported the multilevel results as can be seen in the metrics we presented already in the manuscript including the predictive power (AU value = 96%) compared to the single level (women) multivariable logistic regression results (AU value = 80.4%). Yes, generally the effect sizes and their confidence intervals did not change that much except for household wealth (see rich/richer category for model 1 and model 4). Also, frequency of listening to radio was significant in model 1 but not significant in model 4, suggesting that the standard error was wrongly estimated in model 1, leading to the observed spurious statistical significance. However, this is not the focus of this study as already presented. The focus is on quantifying unobserved household- and community-level differences in the likelihood of health facility delivery among women and our results confirmed that there were significant differences in the likelihood of health facility delivery across households in different communities, an important finding that cannot be achieved using the standard single level binary logistic regression models. We revised the manuscript to provide more details on the multilevel analysis. See lines 529-554 at pages 28-29, lines 571-581 at page 30.

4) As you are making these changes, make these minor edits as well:
- Please only use one number after the decimal (both to be consistent and to not overstate your precision). For example, on page 12, 2.99%->3.0%, but note this is in text and tables.
Response: Thank you. We revised the manuscript to address this throughout the text and tables.

- I still find Table 1 difficult to draw conclusions. Can you change the column percents to be relative within columns (i.e. sum to 100% within each column).

Response: Thank you. These are cell percentages, i.e., the combined columns and rows sum up to 100% to measure contributions across rows and columns simultaneously. Based on your suggestion, we revised the manuscript to have column percentages adding up to 100% as can be seen in the Table 1.

- In conclusion, change cover to coverage.
Response: Thank you. We revised the manuscript to reflect this throughout the manuscript, including the conclusion.

Reviewers' comments:

Review Comments to the Author
Reviewer #1: This manuscript would be a helpful contribution to the knowledge of predictors of facility-based delivery for improved newborn and maternal outcomes in low- and middle-income countries. I hope the authors will find the comments below helpful as they revise the paper.
Response: Thank you for your helpful comments. See point-by-point responses below.

Title:
1) The title may need some improvement for easier reading and understanding. For example: “A multilevel analysis of the predictors of health facility delivery in Ghana: evidence from the 2014 Demographic and Health Survey”.

Response: Thank you. We agreed and revised the title as suggested. See lines 3 and 7 at page 1.

Abstract:
2) In order to help the reader of this paper, you should briefly describe what the Sustainable Development Goal 3 and its target 7 say.
Response: Thank you. We were mindful about the length of the abstract hence our approach. However, we now revised the manuscript to address this in lines 42-44 at page 2

Methods’ section:
3) “Independent variables”: you need to focus on defining your variables (i.e. how GDHS defines each variable and describe any other changes that you might have made to these variables to fit your analyses) and avoid to provide unnecessary details (e.g. numeric codes given to categorical variables) that may confuse readers.

Response: Thank you. We revised to reflect this suggestion. See lines 254-273 at pages 9-10.

4) It’s important that you cited a number of papers that previously studied determinants of facility-based delivery, however it is not clear which conceptual framework you adopted/adapted? There is also a number of potential predictor variables like antenatal care (ANC) services utilization, partner’s education and marital status that you didn’t include in your analysis, why?

Response: Thank you for pointing this out. First of all, the selection of the considered independent variables, including occupation, ANC, partner's education and marital status were informed by the available literature as we earlier presented and cited in the manuscript. Secondly, we examined all these potential predictors of health facility-based delivery and subjected them to rigorous statistical model selection procedures using metrics like the AIC, Log Pseudo-Likelihood and the Area Under Receiver Operating Characteristics (AUROC) Curve in order to arrive at the ‘optimal’ model that is consistent with the data. The set of these covariates in the model that provided the minimum AIC/BIC values and the highest AUROC value were returned in the final model. Thus, only the independent variables retained in the final selected model were presented under the ‘Independent variables’ section. Also, note that the predictive accuracy of our final predictive model interpreted in the manuscript is 96% (see Fig.1 in the manuscript), a value that is very hard to come by in a predictive

modelling of health outcomes in the literature. This clearly suggests that elimination of such few independent variables during the model selection stage could not impact greatly on our final model results interpreted in this study. However, we revised the manuscript to address this. See lines 271-273 at page 10.

5) For the "household wealth index" variable, explain the rationale of collapsing poorer/poorest and richer/richest categories and NOT using the original 5 categories variable as it is provided by GDHS?

Response: Thank you. We adapted this as used in a study from Nigeria (https://journals.plos.org/plosone/article?id=10.1371/journal.pone.0259250#pone-0259250-t001 see Table 1) where they combined the categories 'poorest' and 'poorer' as 'poor', 'richest' and 'richer' as 'rich' and the third category remaining as middle. This also helps to obtain sufficient sample sizes for each category for this variable and to obtain reliable parameter estimates from the fitted model. We revised the manuscript to address this. See lines 268-271 at page 10.

6) Explain why you included in your model the "type of water" and "toilet facility" in the household variables together with the "wealth index" variable, while they are also used in the calculation of the wealth index as a composite measure of the household's economic status.

Response: Thank you. Since the goal is to develop a predictive model to predict health facility-based delivery, inclusion of 'type of water' and 'toilet facility' together with wealth index is acceptable. Also, we examined presence of multicollinearity in our model using generalised variance inflation factor as already stated in our manuscript, and we did not find any evidence of multicollinearity, suggesting that inclusion of these independent variables did not lead to overfitting of the final model presented in this work even though 'type of water' and 'toilet facility' were among several variables considered in the estimation of the wealth index variable.

Results' section:
7) Generally, a final model should only include statistically significant variables, however this seems not to be true for what you reported as final model 4 – please clarify in the methods' section how you arrived at the final models.

Response: Thank you. Respectfully, we disagree with the reviewer that a final model must always include only statistically significant independent variables. In practice and theory, both in prediction and inference, is not only significant independent variables that are retained in the final model. Also, we already provided sufficient evidence in the methods section about how we extend the single-level multivariable model to a two-level and a three-level multilevel models. In arriving at a final model, one must make use of model selection metrics (e.g., AIC, Log Pseudo-Likelihood and the AUROC values) which is part of the results, and we adequately presented these metrics already in the results section under 'Model selection and predictive ability of the fitted models' in lines 402-409 and in Table 2 at page 17-18 and interpreted these in lines 417-427 at pages 18-19. We also stated that the consideration of these independent variables in our models was informed by literature and provided citations for this. See lines 252-254 at page 9.

Discussion section:
8) Please revise the discussion section, and:
a) Include an introductory as the first paragraph for summarizing the study aim and all key findings.

Response: Thank you. We revised to reflect this. See lines 505-554 at pages 27-29

b) Don't repeat the methods and results' sections and focus on interpreting the key findings as well as comparing them with the existing literature.

Response: Thank you. We revised to address this. See lines 512-518 at page 28. Other revisions were made throughout the discussion section for clarity.

Reviewer #2: Please go through the manuscript to clean all grammatical errors, in addition to the comments attached. The manuscript in the current format has several grammatical errors that weaken in it in addition to the points raised in the attached document.

Response: Thank you. We have done extensive proofread to correct possible grammatical errors throughout the manuscript in tracked changes as suggested.

The paper addresses a topical issue on improving skilled birth attendance in Ghana. It looks at factors influencing health facility delivery among pregnant mothers, using the 2014 DHS data. The paper implemented multilevel modelling looking at individual, household and community level factors to predict health facility deliveries. Model performance was addressed using area under receiver operating curve and Akaike information criterion.

Before publishing the paper, the authors need to address a number of points as raised below.
Response: Thank you. We addressed the issues raised point-by-point below:

Major concerns

1. Page 4, the paragraph starting with "the large majority…." The authors present skilled birth deliveries and health facility deliveries as two different components. Can this difference be explained further? In the current format, this is not clear and confusing.
Response: Thank you for pointing this out. We revised the manuscript for clarity by removing the statement. See lines 125-127 at page 4.

2. Page 6, last paragraph, where the purpose of the paper is given. The authors claim that their paper is 'developing a novel multilevel predictive model'. This claim is repeated several times (in the methods, and discussion sections). What is novel about multilevel modelling as used in the current format? Multilevel models as used in the current paper are not novel and therefore this "novelty" claim must be dropped.
Response: Thank you. We revised the manuscript by removing 'novel' from those two places where the word 'novel' was used. See line 202 at page 7 and line 505 at page 27.

3. The paper uses data from 2014, almost 9 years ago. To be more relevant, the authors should consider augmenting this with most recent routine data on health deliveries as so many factors may have changed since the data were collected back in 2014. This also needs to be reported as a weakness of the paper. The paper can be strengthened further once it takes into account of the newer data to understand how SBA and ANC have changed or improved over the years.
Response: Thank you and we appreciate your suggestions. The 2014 DHS data is the most recent full DHS data on this critical topic available in Ghana. Augmenting the results in this study using other routine data is way beyond the scope of this study which we believe the reviewer can appreciate. The reviewer's suggestion could heavily involve submitting application for ethical clearance to the Ghana Health Service, and the individual facilities where this routine data can be obtained, and this can take a lot of efforts and time. As stated earlier, the 2014 GDHS is the most recently available nationwide full DHS survey data available for Ghana on this critical public health issue of global interests. Unfortunately, there is therefore no way out for the authors to use a more relatively recent Demographic and Health Survey (DHS) data on this topic in Ghana. We revise the manuscript to indicate that the 2014 GDHS is the most recent full DHS data available for Ghana. See lines 227-228 at page 8. We have also included a statement in the limitation. See lines 681-682 at page 34. Finally, the data from Ghana DHS round conducted in late 2022 and early 2023 is yet to be released by the implementing agencies and therefore not available for use. We will update our models in our future research on this topic when the new DHS data is made available to the public.

4. What are the specific geospatial mapping approaches used here? Rather, these are just proportions put on a map. Rather than using different colour code for each region, what would be more useful is to put these proportions on a scale of zero (0) to one hundred (100) percent and use a common legend/colour in the scale 0 – 100 for each of the legends. In the current format, the geospatial component is adding very little, if anything to this analysis.
Response: Thank you. We revised the manuscript by deleting this section and proving a summary. in lines 349-351 at pages 13-14 and lines 238-244 at page 9.

Minor concerns
1. Page 8 on independent variables, age has been categorised into ranges: 15 -24, 25 – 34, 35 – 44 and 45 – 49. This lumps adolescents 15 – 19 with young mothers 20 – 24. It would be interesting to look at the outcomes specific to this young group of women 15 – 19 and compare their outcomes to adult women.

Response: Thank you. We adapted the lower age classification of 15-24 used in a study from Nigeria (https://journals.plos.org/plosone/article?id=10.1371/journal.pone.0259250#pone-0259250-t001 see Table 1) and expanded it from their higher class of 35+ to 35-44 and 45-49 in our study. We will consider this suggestion in our future research.

2. Page 8, independent variables, wealth category has been put into 3 categories. Usually, DHS puts this variable into 5 categories namely: poorest, poorer, middle, richer and richest.

Response: Thank you. We adapted this as used in a study from Nigeria (https://journals.plos.org/plosone/article?id=10.1371/journal.pone.0259250#pone-0259250-t001 see Table 1) where they combined the categories 'poorest' and 'poorer' as 'poor', 'richest' and 'richer' as 'rich' and the third category remaining as middle. This also helps to obtain sufficient sample sizes for each category for this variable and to obtain reliable parameter estimates from the fitted model. We revised the manuscript to address this. See lines 268-273 at page 10.

3. Page 9: The authors indicate the following "We applied both single level and multilevel (two and three-levels) binary logistic regression models to analyse data on a total of 4201 mothers residing in 3936 households and 427 communities were analysed with complete measurements on health facility delivery as well as complete measurements on potential predictors considered in the study." The explanation on single level, and multilevel (2 and 3) are currently inadequate and need to be extended to avoid misleading the reader.

Response: Thank you. Thank you. Single-level (level 1 - women) model is just the model with only fixed effects while level 2 model is the one with random effect at the household level and level 3 model is the one with random effect at the community level. We already explained this in the manuscript. However, we revised the manuscript for clarity. See lines 280-281, 287-288 at page 11.

4. Page 9: "For the cluster (i.e., community), the minimum was 1 and the maximum was 33." Are these clusters per district or region?
Response: Thank you. This refers to the number of women in a cluster, suggesting that the minimum and the maximum number of women per cluster was 1 and 33 respectively. See lines 285-286 at page 11.

5. The authors present median odds ratio (MOR) and claim it is easier to interpret. Please define this in clear and simple terms for the reader.
Response: Thank you. We have already stated that it is easier to understand and interpret unlike the VPC because the MOR is on the odds ratio scale. Thus, you will interpret the MOR just as you will do for the usual odds ratios in binary logistic regression models with reference MOR=1 meaning equal odds, MOR>1 meaning increased odds, and MOR<1 meaning reduced odds. We provided interpretation for this in the results and discussion sections. See lines 485-488 at page 26 and lines 532-538 at pages 28-29.

6. Page 13: first line, add % symbols to numbers in parentheses.
Response: Thank you. We addressed this. See line 379 at page 15.

7. Page 27: the authors claim that improved water source in households is a predictive factor for health facility delivery. Was the correlation of this particular variable checked with socio-economic status/wealth status? Rather, this seems to be an artefact of wealth (and education status) that could be driving this outcome.
Response: Thank you. Since the goal is to develop a predictive model to predict health facility-based delivery, inclusion of 'type of water' and 'wealth index' is acceptable. Also, we examined presence of multicollinearity in our model using generalised variance inflation factor as already stated in our manuscript (see lines 344-346 at page 13), and we did not find any evidence of multicollinearity, suggesting that inclusion of these independent variables did not lead to overfitting of the final model presented in this work even though 'type of water' was among several variables considered in the estimation of the wealth index variable.

Reviewer #3: 1 Decision
After performing a careful read I recommend this paper for "acceptance" with some revisions and feel that the content is suitable for readers of the esteemed journal, PLOS Global Public Health conditional on the following comments/corrections being satisfied. The paper still has scope for improvement. Some paragraphs need to be clarified to improve readability. Please see below:
Response: Thank you for the feedback.

2 Major Comments
1. The paper starts with a long introduction. Please divide it into four/five paragraphs. Also, can the abstract be shortened. Being a little more specific and succinct could save a lot of real estate and shorten the paper, making it a better read.

Response: Thank you. We believe that the details presented in the introduction are very important to the study. However, we carried out extensive revisions to address this. See lines 107-192 at pages 4-7. We revised the abstract to address this. See page 2.

2. The authors should clarify what exactly is their methodological contribution. It seems they are just using existing methodologies.

Response: Thank you. This is an application of an advanced multilevel modelling approach to develop a predictive model for a health facility delivery with random effects at household and community levels in Ghana which yielded an excellent predictive power of 96%, a figure very rare to achieve in this area. The goal is to quantify unobserved household- and community-level differences in the likelihood of health facility delivery among women. Thus, we want to answer a question relating to whether households and communities in which women reside can explain the likelihood of these women in the utilization a health facility for delivery. We revised the manuscript to bring this out clearly. See lines 507-511 at page 27 and 532-538 at pages 28-29.

3. Ghana now has 16 regions in Ghana. I think considering the current restructuring will make the paper more appealing to the readers.

Response: Thank you. At the time of the 2014 Ghana Demographic and Health Survey, there were only 10 regions in Ghana. The suggestion of the reviewer can only be addressed when the next survey data from the 2022/2023 Ghana DHS is made available to the public for use.

4. The use of citations in the paper is repetitive, it is generally considered good practice to be more

specific in citations. For instance, under independent variables, you do need 10 references. Consider doing the same for the rest of the paper.

Response: Thank you. Except for references 34 and 35, all others were already cited earlier in the manuscript but for a different purpose. Here, we believe these references could be maintained.

5. On Page 10 out of 36, consider citing as least this paper at least, Liu, Q., & Pierce, D. A. (1994). A note on Gauss—Hermite quadrature. Biometrika, 81(3), 624-629 and mostly explain why you decided to use this approach in your paper.

Response: Thank you. We revised the manuscript to reflect this. We used the mean-variance adaptive Gauss-Hermite quadrature integration approach for likelihood approximation. See line 322 at page 12.

6. In Table 1, can you show both the numbers and percentages under health and non-healthy facility. It will be interesting to see those numbers.

Response: Thank you. We revised to reflect this. See Table 1 at page 15.

7. I think it is better to have the strengths, limitations, and conclusion be under discussion, and provide some recommendations. Again, summarize in less and concise paragraphs.
Response: Thank you. We structured these sections per the journal requirements, and we are happy to keep the current structure. We revised the section to make it more concise. See the discussion section with tracked changes.

3 Minor Comments
1. Page 3 out of 36, space before citing references.

Response: Thank you. We revised to address this. See line 98 at page 3. We did same across the manuscript.

2. First define SDG.

Response: Thank you. Done. See line 108 at page 4.

3. Page 4 out of 36, space before citing references and do same throughout the paper.

Response: Thank you. We revised to address this at line 125 at page 4. We have done this throughout the manuscript.

4. Check the citation under “ethics approval and consent to participate.”
Response: Thank you. We revised to address this. See line 364 at page 14.

Suggested References
Liu, Q., & Pierce, D. A. (1994). A note on Gauss—Hermite quadrature. Biometrika, 81(3), 624-629.

Response: Thank you. We have included this in the manuscript. See line 322 at page 12.
