# Supplementary material for: A multilevel analysis of the predictors of health facility delivery in Ghana: Evidence from the 2014 Demographic and Health Survey
Source: PLOS Glob Public Health. 2024 Mar 13;4(3):e0001254. doi: 10.1371/journal.pgph.0001254 (PMC10936823; doi:10.1371/journal.pgph.0001254)
Supplement: Attachment — Submitted filename: Rebuttal letter R2.pdf [file pgph.0001254.s003.pdf]

A multilevel analysis of the predictors of health facility delivery in Ghana: evidence from the 2014 Demographic and Health Survey

POINT-BY-POINT RESPONSES TO EDITORS AND REVIEWERS

We wish to express our heartfelt thanks and appreciation to the Editors and the Reviewers for taking time of their tight schedule to review our manuscript and we found their comments very useful.

We have taken all the necessary steps to respond to your comments and thus, resubmit the revised manuscript for consideration and subsequent publication in your cherished journal.

The responses to your comments are presented below and the changes effected in the main manuscript for your consideration. Furthermore, minor changes have been made to the manuscript purposely to enhance the quality of the manuscript.

Journal Requirements:

1. Please provide additional details regarding participant consent. In the ethics statement, please ensure that you have specified what type you obtained (for instance, written or verbal, and if verbal, how it was documented and witnessed). If your study included minors, state whether you obtained consent from parents or guardians.

Response: We have already provided this in the main manuscript under the subheading "***Ethics approval and consent to participate***" at page 12 and provided citation to support this. See lines 305-314. As a result, we did not make any additional changes to the manuscript.

Please review your reference list to ensure that it is complete and correct. If you have cited papers that have been retracted, please include the rationale for doing so in the manuscript text, or remove these references and replace them with relevant current references. Any changes to the reference list should be mentioned in the rebuttal letter that accompanies your revised manuscript. If you need to cite a retracted article, indicate the article's retracted status in the References list and also include a citation and full reference for the retraction notice.

Response: We have done this, and they are complete. We did not find any of them retracted. We did not make any changes to the manuscript.

Additional Editor Comments (if provided):

Overall, I and the reviewer found that you were responsive to the initial comments. We have some additional comments, mostly minor.

Response: Thank you.

More substantive edits:

- Please walk back the statement: "... it guarantees a reduction in complications during and immediately after delivery, a safe birth, and maximises the survival chances..." This is an overstatement of what we know and what we can conclude about giving birth in facilities.

Response: We revised the manuscript to address this. See lines 114-116 at page 4.

- Remove all of the mapping references, including the map itself. It really doesn't add, and in my opinion detracts from the paper.

Response: We have removed the maps and the references. See lines 190-193 at page 7, lines 300-302 at page 12, lines 431-440 at page 23.

- Your last sentence of Independent variables: "... but these were removed during model selection..."? Say more, why? What was the process? Your first table you show everything is significant, is this because you removed the insignificant factors? This needs to be clarified, and most likely moved to the analysis section.

Response: First, we identified potential predictors of health facility delivery or skilled birth attendance based on the available literature which we already stated in the manuscript. However, it is possible that a wholesome inclusion of all these identified covariates could lead to the overfitting of the multivariable logistic regression models so to address this, we used both backward elimination and forward selection procedures to identify the sets of predictors that could provide a good fit to the data. We revised the manuscript to address this. See lines 220-225 at page 8. Also, note that we had an excellent predictive power of 96% (i.e., 96 percent correct classification) in our final model (Table 2 and Figure 1). Also, the significance of all the predictors in Table 1 is possible because these are just crosstabulations between the health facility delivery status and each of the predictors with p-values based on Pearson Chi-square statistics as already presented in the footnote of Table 1. Thus, we did not adjust for all these predictors simultaneously in Table 1 because that is not possible as in the case of the multivariable logistic regression model presented in Table 2 where not all predictors were significant in the multivariable logistic regression models.

- Results - you keep jumping tenses. This should probably be in past tense. More than anything, be consistent. (It is throughout the results, but for example, "We then extended model 1..."

Response: We revised the manuscript to address this throughout the results section. See pages 13-17.

- Doesn't Model 4 have household and community factors. Is it not possible to calculate the household-level VPC and MOR? (see table). Very possible this cannot be done, and if so, then be sure to add a footnote.

Response: We have computed this and included it in Table 3. Also, see lines 426-427 at page 23.

- One comment you did not address here - in the limitations, you say this is the most recent data. Do you think these results will hold the test of time? Still relevant now?

Response: We provided further details in the limitation to address this. See lines 528-531 at page 27.

- One comment you did not address here - in the conclusion, you say "We therefore propose a household..." which households/communities? ANd what interventions? (I don't think you can say what interventions based on what is here, but maybe you can... if not, then you need to say this has to be studied.

Response: We revised the manuscript to address this. See lines 537-539 at page 27.

Minor edits:

- In the introduction, change to STD to STG (STD target 3.7)

Response: Revised to SDG target 3.7. See line 100 at page 3.

- Remove the commas in this sentence to read: "Though maternal and child healthcare and delivery services are free, ..."

Response: Done. See line 142 at page 5.

- Add a comma to "These include maternal education (categorized as no education, primary....)

Response: Done. See line 203 at page 8.

- Edit to ... nested within household which are nested...

Response: Done. See line 241 at page 9.

Review Comments to the Author

Reviewer #3:

1) How did you select the independent variables? Need some clarifications.

Response: We used both backward and forward selection procedures. We revised the manuscript to address this. See lines 220-225 at page 8.

2) How was missing data handled?

Response: As already presented in the manuscript, the analysis is based on complete measurements on health facility delivery and potential predictors (i.e., listwise deletion). See line 233-235 at page 9.

3) Space before citing references, see Page 4 of 31 among others.

Response: We revised to address this throughout the manuscript.

4) For consistency it will be best to represent all confidence intervals as (0.2-0.5) or (0.2, 0.5). Just stick to one of these. Also, space out number (percentage) in Table 1.

Response: We revised accordingly throughout the manuscript, including the abstract using the (0.2-0.5) format.
